# Research priorities for measuring biologic age: summary and future directions from the Research Centers Collaborative Network Workshop

**DOI:** 10.1007/s11357-022-00661-w

**Published:** 2022-10-15

**Authors:** Tina E. Brinkley, Jamie N. Justice, Shubhashrita Basu, Scott R. Bauer, Kah Poh Loh, Peter Mukli, Ted Kheng Siang Ng, Indira C. Turney, Luigi Ferrucci, Steven R. Cummings, Stephen B. Kritchevsky

**Affiliations:** 1 Department of Internal Medicine, Section on Gerontology and Geriatric Medicine, Wake Forest University School of Medicine, Winston-Salem, NC USA; 2 Center for Demography of Health and Aging, University of Wisconsin, Madison, WI USA; 3 Departments of Medicine and Urology, University of California and San Francisco VA Medical Center, San Francisco, CA USA; 4 Division of Hematology/Oncology, Department of Medicine, University of Rochester Medical Center, James P. Wilmot Cancer Institute, Rochester, NY USA; 5 Department of Biochemistry/Molecular Biology, University of Oklahoma Health Sciences Center, Oklahoma City, OK USA; 6 Edson College of Nursing and Health Innovation, Arizona State University, Tempe, AZ USA; 7 Department of Neurology, Columbia University Medical Center, New York City, NY USA; 8 Intramural Research Program of the National Institute On Aging, NIH, Baltimore, MD USA; 9 San Francisco Coordinating Center, California Pacific Medical Center Research Institute and the Department of Epidemiology and Biostatistics, University of California, San Francisco, CA USA

**Keywords:** Biologic age, Aging, Epigenetics, Clinical trials, Multidisciplinary studies

## Abstract

Biologic aging reflects the genetic, molecular, and cellular changes underlying the development of morbidity and mortality with advancing chronological age. As several potential mechanisms have been identified, there is a growing interest in developing robust measures of biologic age that can better reflect the underlying biology of aging and predict age-related outcomes. To support this endeavor, the Research Centers Collaborative Network (RCCN) conducted a workshop in January 2022 to discuss emerging concepts in the field and identify opportunities to move the science forward. This paper presents workshop proceedings and summarizes the identified research needs, priorities, and recommendations for measuring biologic age. The highest priorities identified were the need for more robust measures, longitudinal studies, multidisciplinary collaborations, and translational approaches.

## Introduction

Aging is characterized by molecular and cellular changes that generally lead to the accumulation of cellular and tissue damage and physiological dysregulation [1]. When damage surpasses reserve and resilience capabilities, the onset of disease can occur, along with loss of function, subsequent frailty, and ultimately death. Although lifespan has increased over the last 15 years due to decreasing mortality rates, there has not been a matching increase in health span. In fact, chronic complex morbidity is on the rise, and years of life lived with disease have increased. However, there is significant heterogeneity in phenotypic aging consistent with the varying rates of biologic aging and gene expression profiles across cells, tissues, and organs, both within and across individuals [2, 3]. Moreover, late-life diversity in the aging rate likely arises through stochastic effects of genetics, epigenetics, and environmental factors, suggesting that the aging process is not immutable. As such, there is an urgent need to better understand the molecular and cellular changes that contribute to functional decline with advancing age, as interfering with the basic fundamental mechanisms may reduce the global susceptibility to age-related chronic diseases [4].

Conventionally aging has been quantified using a simple measure of chronological age, which is the number of years, months, days, or hours since birth. However, chronological age fails to account for the heterogeneity with which individuals age over time. Thus, biologic age has been proposed to be a more accurate measure of aging that reflects the impact of various exposures (e.g., genetic, lifestyle, environmental) across the life course that may slow or accelerate physiological mechanisms of aging. Measures of biologic age also provide the foundation for evaluating treatments designed to affect the aging process, which is the fundamental risk factor for age-related diseases. Elucidating the determinants of biologic aging and its rate and figuring out how to measure it accurately are critical issues in the emerging field of geroscience and are key to identifying robust and valid measures of biologic age [4, 5].

In January 2022, the Research Centers Collaborative Network (RCCN) sponsored a 1.5-day virtual workshop on Measuring Biologic Age. The scope of this workshop included sessions on defining biologic age, identifying relevant markers of physiological aging, evaluating the utility of aging biomarkers, and understanding potential applications and implications. The agenda, recordings, and slides are archived at www.RCCN-aging.org. This paper describes the proceedings, discussion, insights, and recommendations that came out of this important workshop, including a list of research needs, priorities, and opportunities on the issue of measuring biologic age.

## Understanding biologic mechanisms of aging

To develop suitable measures of biologic age, it is essential that we understand the mechanisms contributing to the aging process. The seminal paper by Lopez-Otin et al. in 2013 characterizing nine candidate cellular and molecular hallmarks of aging has helped organize efforts in understanding, quantifying, and utilizing measures of biologic age in research [6]. These hallmarks describe primary causes, resilience mechanisms, and secondary consequences that are generally considered to contribute to the biologic aging process and collectively determine the aging phenotype. For example, cellular senescence is triggered by molecular damage and metabolic stressors that lead to aberrant cell cycle activity. The resulting stable cell cycle arrest and senescent phenotype is highly metabolically active and includes altered morphology, gene expression, and a pro-fibrotic and pro-inflammatory secretome (i.e., senescence-associated secretory phenotype (SASP)) [7, 8]. Genomic instability leads to the accumulation of genetic damage that alters the integrity and stability of DNA (both nuclear and mitochondrial), partly due to increases in somatic mutations and insufficient DNA repair mechanisms. Epigenetic changes involving alterations in DNA methylation patterns, post-translational modification of histones, and chromatin modeling can impact gene expression. Finally, altered intercellular communication driven by inflammaging and immunosenescence [9, 10], among other pathways, promotes interorgan coordination of the aging phenotype as age-related changes in one tissue can lead to deterioration in other tissues. In the following section, we describe various measures of biologic age that have been developed to reflect these mechanistic hallmarks of aging.

## Measuring biologic age

A variety of approaches have been developed to quantify the biologic aging process, some of which estimate biologic age using mathematical algorithms applied to specific indices such as DNA methylation patterns (epigenetic clocks) or alterations in the proteome (proteomic clocks). Other methods yield signatures of biologic aging that reflect characteristic markers of key cellular and molecular processes (e.g., senescence, somatic mutations). In addition, composite measures that incorporate multiple indices across multiple levels have also been developed to reflect the combined contribution of biologic mechanisms leading to the aging phenotype.

Epigenetic clocks estimate the age of tissues and cells using multivariate weighted sums of CpG sites in the genome (i.e., epigenetic age). To date, more than a dozen epigenetic clocks have been developed including the Horvath 1 and Horvath 2 [11], the Hannum [12], and the Lin [13] clocks. Although one of the biggest strengths of these first-generation clocks is their ability to predict chronological age systematically across all human cell types and tissues, they are based solely on information from CpG methylation. To better capture the underlying biologic features of accelerated epigenetic aging, second-generation clocks, including DNAm PhenoAge [14] and DNAm Grimage [15], incorporate more complex phenotypes based on mortality risk. More recently, the DunedinPoAm clock was developed to capture within individual variation in rate of aging over time [16]. However, all epigenetic clocks have a signal (biologic variation) vs. noise (technical variation) problem, in part because data from individual CpGs are often noisy and unreliable [17, 18]. To reduce technical variability, Higgins-Chen el al. proposed using principal component analysis (PCA) rather than individual CpGs to train the clocks [17]. The main benefit of this method is that PCA can extract the shared aging signal across intercorrelated CpGs while ignoring the noise. This method not only generates better reliability and a steadier age change gradient, but also reduces the sample size needed for clinical trials. PCA-based clocks are now used for clinical trials, longitudinal tracking and cell culture [17], single cell epigenetic clocks [19], Pan-mammalian clocks [20], ultra-low-cost epigenetic clocks [21], and DNA methylation editing with CRISPR [22]. While PCA-based clocks work well in characterizing groups, it is less clear how well these clocks perform in individuals followed through time or in response to interventions.

Proteomic clocks are also mathematically derived estimators of biologic age based on the characterization and quantification of proteins. Recent technological advancements, including mass spectroscopy-based proteomics and multiplexed proteomic assays using modified aptamers (SOMAscan) and proximity extension assay (PEA, O-Link), permit assessment of thousands of proteins in plasma or other specimen types to yield new clinical biomarkers. These proteomic clocks (such as the one based on SOMAscan) reflect chronological age and have been used in large cohort studies, including the Baltimore Longitudinal Study on Aging (BLSA), Genetic and Epigenetic Signatures of Translational Aging Laboratory Testing (GESTALT), and “Invecchiare in Chianti” (InCHIANTI), with > 80% replication across cohorts [23, 24]. Moreover, proteomic clocks are used to identify persons who are predicted to be “older” than their chronological age based on their proteomic signature. These “age-accelerated” people have a higher risk of mortality and faster accumulation of disease [25]. A strength of proteomics versus other data-intensive omic platforms is that select proteins—alone or in combination—can be identified and inform mechanistic understanding of biologic aging. In contrast, many earlier-generation epigenetic clocks are powerful predictors of chronological age, yet the relevance to phenotypic age may be difficult to discern. Indeed, as proteomic platforms become more accessible, meta-analyses across diverse human cohorts will be necessary to provide convergent evidence for key protein nodes that change with chronological and biologic age [26, 27]. Collectively, these advancements in proteomic biomarkers of aging underscore their utility and promise, though technical and analytic refinements remain.

Cellular signatures of senescence help advance our understanding of what and how to measure cellular senescence in vivo. Many cell types can acquire a senescent phenotype, and there are many cellular stressors, oncogenic signals, and sources of molecular damage that can initiate cell senescence and present an in vivo signature of senescence [28, 29, 30]. There is also considerable cell-type heterogeneity using single marker genes as surrogates for “senescence” [31, 32]. This heterogeneity is one of the defining challenges for senescence-derived biomarkers as there is no “universal” marker for identification of senescent cells in vivo. The emergent view is that a panel of biomarkers and tissue-specific maps may be necessary to identify heterogeneous senescent cells [33, 34]. For example, novel bioinformatic approaches are being used to develop tissue-specific maps of senescent cells in the brain. Recent investigations constructed unbiased composites of multiple genes—called senescence eigengenes—that each represents distinct aspects of senescence, including stress response, cell cycle arrest, and inflammatory response [35]. This allows high-resolution mapping of cells and cell environments and uses hypothesis-informed validated biomarkers of senescence. This innovation represents a new and powerful approach to understanding the mechanisms governing cellular senescence which can guide potential treatments for diseases such as Alzheimer’s disease (AD).

Blood signatures of senescence are being developed using a similar multianalyte approach, which is a critical area of development as accessible biospecimens are essential for clinical translation [36]. Recently, a “SASP Atlas” was constructed based on a comprehensive proteomic analysis of soluble and exosome SASP factors [28]. The SASP profile was derived from multiple senescence inducers and cell types yielding hundreds of proteins. Interestingly, the secretome of senescent cells demonstrated considerable overlap with plasma biomarkers of biologic aging [24]. New methods in profiling using advanced proteomic approaches based on unbiased data-independent acquisitions (DIA) mass spectrometry (MS) have expanded identified SASP factors by about tenfold and can be applied to comprehensively and quantitatively profile exosomes released from senescent cells [37]. Though research on exosomes is challenged by their isolation and analysis in circulation, these can be overcome using DIA-MS proteomics and multiomic workflows. Collectively, multiomics of soluble and exosome SASP are novel tools to explore the role of cell senescence in human aging and as therapeutic targets. However, a key challenge to identifying and interpreting SASP factors in the blood is the lack of specificity. Many SASP factors are important signaling factors that represent (patho)physiological processes that are not related to cellular senescence per se.

Mutational signatures are characteristic combinations of mutation types arising from specific mutagenic processes such as DNA replication infidelity, defective DNA repair pathways, and DNA enzymatic editing. Although these signatures have been used predominately in the oncology field to characterize the accumulation of somatic mutations in various cancers, somatic mutations likely contribute to the aging phenotype as well. However, solid evidence for this is lacking. Multiple studies demonstrate that somatic mutation frequency is negatively correlated with lifespan across multiple species, irrespective of maximum lifespan, and report a positive correlation between mutation frequency and age [38, 39, 40]. Using single-cell approaches, researchers can now characterize the landscape of somatic mutations with aging in humans [41]. The creation of the “SomaMutDB” database that documents the 2.5 million mutations discovered in normal somatic human tissues will further advance the field [42]. Studying extreme phenotypes, such as centenarians and progeroid syndromes, can also help understand the role of genomic instability and DNA damage. On one hand, progeroid syndromes are caused by specific genetic mutations that lead to premature aging in one or more organs. On the other hand, centenarians are a model of successful aging or delayed biologic aging who not only live longer lives, but also experience comorbidities for a shorter amount of time (unlike most older adults who spend 10–20 years of life with age-related diseases and disability) [43]. Studying the role of affected genes (e.g., lamin A, WRN) in the case of progeria or genetic signatures of longevity in centenarians and their offspring can provide useful information on molecular mechanisms of aging, including biomarkers that may be predictive of mortality and/or resilience [44, 45, 46, 47, 48].

Composite measures are based on various combinations of biologic clocks or clinical indices and reflect the complex effect of aging on different physiological systems (e.g., renal, cardiovascular, endocrine, immune, pulmonary) [5, 49, 50, 51]. Such composites are often used in studies that incorporate age-normalized physiology, an approach that defines biologic age as the age at which a person’s biology would be “normal” in a reference population and defines “pace of aging” as the rate of decline in integrity across multiple organ systems [52]. While this approach is useful for identifying individuals with a greater than expected biologic age or rate of phenotypic aging and an increased risk of age-related diseases, disability, and mortality [5, 53], defining biologic age in this way requires several additional considerations. Notably, the reference sample used to define “normal” biologic age should reflect the distribution of causes and features of aging for the target population in which the measurement is used. The level of analysis used to compare the aging biology (e.g., cellular, organ/organ systems, physical function, disease counts) and the unit of time for defining biologic age (e.g., time since birth, time until death, coordination across biologic systems) are also critical factors, yet the optimal measures remain unknown. In addition, the minimum timescale over which natural aging and intervention-modified aging can be observed in human longitudinal studies is also unclear. Despite these challenges, recently published data from the Baltimore Longitudinal Study of Aging show that annualized change in a global longitudinal phenotypic metric of aging is associated with multiple aging outcomes, including physical and cognitive decline, multimorbidity, and mortality [54]. These findings demonstrate the potential utility of a composite measure to reflect the aging process, although it remains untested whether this measure is indeed modifiable.

## Incorporating measures of biologic age in clinical studies

Geroscience reflects the intersection of basic aging biology and chronic disease and health, linking cellular and molecular changes to global declines in organ function and the development of physical and cognitive impairments [55]. In this regard, validated measures of biologic age are indispensable for tracking age-related changes in function or measuring the efficacy of planned interventions. The aging phenotype depends on multiple factors such as genetics, nutrition, exercise, sex, and lifelong exposure to different pathogens. This evidence lays the groundwork for identifying modifiable factors via epidemiological studies with long-term follow-up and randomized clinical trials with nutritional, lifestyle, or pharmacological interventions that precisely target the relevant pathophysiological process [56, 57]. With this approach, we can say that we have slowed phenotypic aging if a change in the underlying biology affects the clinical phenotype and functional measures of aging. Unfortunately, there are only a few ongoing studies currently employing translational geroscience approaches in multisite clinical trials, including the Molecular Transducers of Physical Activity Consortium (MoTrPAC) and the Metformin for Prevention of Frailty in Older Adults study [58, 59]. Therefore, establishing a causal relationship between biologic and phenotypic aging in humans is a critical next step for future studies.

The concept of geroscience is not disease-specific, but rather implies shared mechanisms and common pathophysiological processes underlying age-related diseases and conditions. Accordingly, investigations that move beyond disease-centered models are necessary to clarify the role of biologic aging in the development of age-related functional decline. As an example, frailty and physical function are person-centered outcomes that integrate multiple pathologies that could be affected by biologic mechanisms of aging, reflect reserve capacity, and strongly predict key clinical outcomes [60, 61, 62]. Notably, there is considerable overlap between the cellular hallmarks of aging and the proposed biologic mechanisms of frailty and physical function. In animal models, the hallmarks of aging appear to contribute directly to frailty [63]. Although data are much more limited in humans, there is a growing body of observational data linking frailty and physical function to biologic mechanisms. Frailty and physical function are also increasingly being used as primary outcomes in randomized clinical trials of interventions targeting biologic mechanisms of aging. While some studies have demonstrated efficacy in improving or preventing declines in physical function [64, 65] or decreasing the rate of aging [66, 67], no interventions targeting frailty itself have demonstrated efficacy. The findings of future trials will be critical to demonstrating that frailty is modifiable and that biologic mechanisms of aging cause frailty in humans [60]. For a more in-depth discussion of how to validate surrogate endpoints in clinical trials, see the accompanying commentary by Cummings et al. [68].

## Moving the field forward

Measuring biologic age allows researchers to characterize the physiology of aging, evaluate how exposures through life affect the aging process, validate potential surrogate endpoints in trials to discover new treatments and intervention targets, and potentially improve healthcare decisions. However, we need to better understand what makes people age differently considering genetic inheritance, individual health-related behaviors, healthcare access, and environmental exposures over the life course. Moreover, several key questions remain, including the following: Are current measures of biologic age sufficient? What information would it take to demonstrate this? Do we need different measures of biologic age depending on the research or clinical setting? How well do measures of biologic age correlate with clinical measures? Below, we address major gaps in the field, highlighting the research needs, priorities, and opportunities to move the field forward.

- *Clarify mechanisms and effects of biologic aging*: Although some measures of biologic age can provide a comprehensive assessment of aging features and predict various age-related outcomes, it is sometimes unclear what exactly is being measured (e.g., inflammation, metabolic function, DNA damage) [69]. To this end, we need to better understand the biologic processes and mechanisms that occur alongside changes in measures of biologic age and establish causal impacts rather than associations. Since each “level” or type of biomarker conveys different information about biologic aging, the best type of biomarker(s) to use in a measure of biologic age also needs to be elucidated. These unresolved issues highlight the need for measures of biologic age that are standardized, easy to measure, sensitive to change (even in younger and healthier groups), clinically meaningful, and easily implemented in either clinical or research settings.
- *Clarify the utility of biologic aging clocks*: Many different clocks have emerged that reflect different biologic mechanisms or hallmarks of aging. Some were meant to estimate biologic age using one marker at a single time point, whereas others use a composite index of multiple tissue-/organ-specific clocks across time. Some were developed to predict chronological age, whereas others were developed to predict aging phenotypes (e.g., frailty). This heterogeneity, coupled with the poor correlation between clocks, suggests that each clock represents a different profile of biologic determinants [49]. It is also possible that clocks are simply stochastic measures of aging (reflecting random accumulation of molecular changes over time) rather than true measures of biologic processes of aging. Moreover, the best way to operationalize biologic aging clocks is also uncertain, and basic information like the within-person reproducibility and analytic variation of these measures is only now being worked out. This has important implications for designing and evaluating trials that target specific pathways reflected by the different clocks.
- *Enhance methodological rigor*: To develop validated measures of biologic age, we must first improve the breadth and depth of our measurements. For example, methodological hurdles still make it challenging to examine cellular components of immunity and other biomarkers that are technically difficult to measure [70]. An incomplete evaluation, and therefore understanding, of biologic mechanisms can also confound our interpretation of disease vs. resilience pathways, as has been suggested in brain aging [71, 72, 73, 74]. To overcome these limitations, we should seek to capitalize on recent technological advancements in multiomic approaches and move beyond blood to incorporate a diverse array of relevant cells, tissues, and other biospecimens. This will be necessary to provide robust measures that accurately characterize biologic aging.
- *Increase the number of longitudinal studies*: The first generation of studies trying to calculate biologic age was cross-sectional. There are well-cataloged biases in inferring longitudinal relationships from cross-sectional data. Moreover, with respect to biologic age, the fact that someone has aged more quickly in the past—the information that cross-sectional data provides—does not necessarily mean that person will continue to age more quickly in the future. Longitudinal studies are ideal for characterizing biologic aging processes (e.g., mechanisms of accelerated aging) because they can account for changes and differences in lifetime exposures across different groups and life course stages. This is critical for assessing relationships over time to determine critical time points for intervention (e.g., early childhood, midlife). Longitudinal studies can also overcome challenges in modeling mortality or loss to follow-up, especially as this varies across diverse groups of people (e.g., race/ethnicity, sex/gender). This study design can be used to address important unresolved questions like: When in the human life course does aging begin? Does the rate of aging vary across the lifespan, and if so, how? Are different methods to study aging needed at different life course stages? Currently, there are too few longitudinal studies in humans to look at rates of changes in biologic age, and those that do exist generally begin at age 20 or older. Thus, there is a need for longitudinal studies across the life course to study multiple measures of biologic age from various organs and cells, how they are connected to each other, and how they are related to development, environmental exposures, and responses to stressors.
- *Identify outcomes for geroscience-focused clinical trials*: Part of the promise of measuring biologic age is in developing and validating surrogate endpoints for trials testing interventions that attempt to affect the aging process to slow or prevent the emergence of age-related health conditions. In the context of clinical studies, measures of biologic age can also be potentially valuable tools for population stratification, inclusion into intervention/clinical trials, and patient selection (e.g., identifying those who may benefit most from an effective geroscience intervention). To do so, we must first identify surrogate markers that fit in the pathway of the treatment’s biologic mechanism of action and predict both clinical outcomes and the effects of the treatment on these outcomes. This issue is discussed at length in the accompanying commentary by Cummings et al. [68].
- *Encourage multidisciplinary collaborations*: Existing NIA networks and center programs such as the Translational Geroscience Network and the Predictive Biomarker Network should be engaged to develop standardized measures and methods across research teams, allowing the pooling of study populations and results. The integration of insights from the aging biology into other fields like economics, sociology, and psychology should also be promoted. For example, quasi-experimental techniques (e.g., instrumental variables, differences-in-differences, and regression discontinuity) can be used to simulate randomized controlled trial designs to examine causality and capitalize on the usefulness and availability of biomarker/molecular data in large population-based cohorts focused on aging. To enable cross-disciplinary and multidisciplinary collaborations and studies, more advanced statistical modeling approaches such as latent variables, multiple moderation, and mediation modeling could be employed. In addition, systematic interactions between researchers working at different stages of the life course will be important for harmonizing relevant data collection and specimen collection.
- *Incorporate social determinants of health*: There are few epidemiological studies linking socioeconomic measures with biologic aging markers across the life span. Thus, more studies are needed to better understand how social determinants of health (i.e., life course sociocultural and structural measures) “get under the skin” and impact biologic aging. Measuring biologic age across and within populations will help to inform our understanding of shared biologic processes in diverse groups of people. When investigated alongside contextual factors, such studies may also shed light on biologic aging as a potential mechanism to explain health disparities. For example, exposure to stress, social disadvantage, structural racism, and discrimination may cause a faster pace of aging, premature decline in health, and racial/ethnic disparities in late-life diseases among older non-Hispanic Black and Hispanic individuals. Moreover, examining social determinants of biologic aging could provide interdisciplinary evidence to potentially inform public policies.
- *Integrate measures of resilience*: Much of the current research has focused on documenting damage accumulation over time. Although resilience is also a key feature in the model of intrinsic aging, its role in delaying or preventing phenotypic manifestations of advanced biologic age, such as frailty or impaired physical function, remains unknown. Importantly, compensation and resilience affect the temporal relationship between various aging metrics. Thus, by integrating accurate and predictive measurements of resilience, we may elucidate how these measures can be adapted to benefit biologic age. In addition, stress or “resilience” tests may help identify individuals with accelerated aging, subclinical disease, or impaired physical function.
- *Utilize a translational approach*: Investigations of biologic age need to be developed across the translational spectrum. For example, understanding the biologic mechanisms of aging will require hypothesis-driven experimental manipulation in cell cultures. Longitudinal studies in clinically relevant animal models of human aging are warranted to better understand the aging process and to assess the validity of aging biomarkers and the usefulness of candidate interventions. As discussed previously, epidemiologic studies, including randomized clinical trials, are needed to translate preclinical findings and validate relevance of biologic aging measures in humans, as there is currently a disconnect between advances made in animal models and human studies. Replicating measures and findings across species and tissues (e.g., blood, urine, cells) will be critical. Although clinical studies have informed aging phenotypes and predictors (e.g., biomarkers for disability, frailty, and dementia), it remains unknown whether these biomarkers can similarly predict health span, thus warranting further investigations. Ultimately, it will be necessary to establish the societal implications of these findings, where the proper and ethical use of biologic age in public health must be determined.

## Conclusions

In sum, there are numerous advantages to measuring biologic age. These measures have the potential to provide an endpoint that reflects underlying aging biology, including subtle changes in seemingly healthy people with accelerated aging prior to physical manifestations. By providing insight into exposures and life experiences, measures of biologic age can be used to track life course effects (i.e., positive and negative events throughout life) and better characterize heterogeneity in phenotypic aging. This information can ultimately help to identify risk and protective factors and determine how to mitigate the impact of adverse exposures. If properly validated, measures of biologic age can be used in clinical studies, e.g., to inform patient selection, risk stratification, and timing of interventions. Notably, some measures are sensitive enough to detect large effect sizes and reduce measurement error, negating the need to employ large samples to identify treatment effects, especially in older surviving samples. Although we are far from being able to use these measures in the healthcare setting, biologic age is a promising tool with potential applications that can ultimately revolutionize the field of aging and significantly impact the care of our rapidly expanding older adult population.
